# INSPIRE: Inclusivity in plastic surgery – Insights, recommendations, & education. A national cross-sectional study exploring perceptions of mentorship among aspiring plastic surgeons

**DOI:** 10.1016/j.jpra.2026.07.020

**Published:** 2026-07-29

**Authors:** Garikai Kungwengwe, Umar Rehman, Simon Filson, Elena Whiteman, Ibrar Perwaiz, Mohammad Sohaib Sarwar, Benjamin Cook, Karanjot Chhatwal, Sammy Arab, Tiffanie-Marie Borg, Manaf Khatib, Naveen Cavale, Peter Brennan, Farah Bhatti, UKPRC Collaborators

**Affiliations:** a Imperial College Healthcare NHS Trust, London, UK; b Division of Surgery and Interventional Science, University College London, London, UK; c Department of Plastic Surgery, Evelina Hospital, St Thomas’ Hospital, London, UK; d Chelsea & Westminster Hospital, London, UK; e Department of Medicine, Bradford Royal Infirmary, Bradford, UK; f Department of Oral and Maxillofacial Surgery, Bradford Royal Infirmary, Bradford, UK; g Faculty of Life Sciences and Medicine, King’s College London, London, UK; h Imperial College School of Medicine, London, UK; i Department of Plastic Surgery, Salisbury Hospital, UK; j Lister Hospital, East and North Hertfordshire NHS Trust, UK; k King’s College Hospital and Guy’s & St Thomas’ Hospitals, London, UK; l Department of Oral and Maxillofacial Surgery, Queen Alexandra Hospital, Portsmouth, UK; m Department of Cardiothoracic Surgery, Swansea Bay University Health Board, UK

**Keywords:** Plastic surgery, Mentorship, Underrepresented groups, Socioeconomic barriers

## Abstract

**Background:**

Plastic surgery is a highly competitive surgical speciality in the United Kingdom. Barriers to entry disproportionately affect ethnic minority candidates. Limited mentorship, unequal access to research opportunities, and systemic biases contribute to differential attainment and hinder career progression.

**Methods:**

A national cross-sectional survey was conducted amongst UK medical students and pre-specialty doctors to explore perceptions of mentorship, access to research, and clinical exposure in plastic surgery. A 20-item questionnaire was distributed via social media and institutional networks. Binary, multinomial, and ordinal logistic regression were used to identify predictors of plastic surgery interest and career progression.

**Results:**

Among 2528 respondents, 20.5% (n = 517) expressed interest in plastic surgery, with comparable interest between men (26.4%) and women (21.4%). Completing a plastic surgery rotation more than doubled interest (adj.OR 2.11, 95% CI 1.55–2.86, p < 0.001), and research involvement nearly quadrupled interest (adj.OR 3.95, 95% CI 2.70–5.78, p < 0.001). Mentorship significantly increased research engagement (adj.RRR 9.20, 95% CI 6.31–13.40, p < 0.001). Despite comparable interest, men were more likely than women to progress to speciality training (OR 1.70, 95% CI 1.24–2.35, p < 0.001). Ethnic minority candidates had lower progression rates (OR 0.43, 95% CI 0.31–0.61, p < 0.001). Mentorship reduced perceptions of racial (adj.OR 0.84, p = 0.048) and gender bias (adj.OR 0.78, p = 0.010).

**Conclusions:**

Structured mentorship is strongly associated with increased research involvement, speciality readiness, and confidence among aspiring plastic surgeons. Its protective effect against racial and gender bias underscores the need for targeted mentorship to promote diversity and equity in plastic surgery training.

## Introduction

The National Health Service is described as 'more diverse than ever',^1^ yet access to specialty training remains inequitable. Plastic Surgery (PS), a highly competitive field, offers limited structured exposure at undergraduate and postgraduate level, leaving many reliant on self-directed learning and extracurricular credit to improve selection prospects.^2^ However, access is often shaped more by sociodemographic factors than ability alone.^3^ Identity-concordant mentorship, a key driver of clinical and academic engagement, remains inconsistent, highlighting the need for targeted widening access initiatives to ensure talent is cultivated on merit rather than constrained by circumstance.^4^

Baroness Helena Kennedy’s diversity report (2021) for the Royal College of Surgeons of England (RCSEng) called for a surgical workforce reflective of the population it serves.^5^ Despite this, the diversity of the PS workforce remains uneven, and its true extent depends on career stage and on the data source examined. Earlier analyses of NHS Digital workforce records reported that the share of reconstructive PS consultants from some EM groups was low and, for certain groups, declined between 2009 and 2020, with slower growth in Asian representation (17–20%) than in comparator specialties (20–29%).^6^^,^^7^ More recent and comprehensive workforce data present a more nuanced picture. The BAPRAS Workforce Planning Survey, the most complete dataset for the UK and Republic of Ireland (100% response rate), together with figures reported at the 2025 BAPRAS Annual General Meeting, indicates that EM surgeons now account for approximately 31% of the consultant body (60% White, with 9% undisclosed). Differences between these sources reflect variation in ethnic classification, completeness of self-reported data, and the populations sampled, and should be interpreted with caution.^8^ At entry to higher surgical training, by contrast, EM candidates are well represented. Of 230 applicants to PS ST3 in 2023, EM candidates comprised 47% (n = 108) and received 49% of offers (n = 51), compared with White applicants at 41% (n = 94) and 42% of offers (n = 44); ethnicity was not stated for a further 12% (n = 28). Offer rates were therefore broadly comparable across groups (approximately 47% of both EM and White applicants received an offer, against an overall rate of 45%), and EM representation at this stage exceeds the 18% EM share of the UK population recorded in the 2021 Census. Under-representation is most apparent later in the pathway, in progression to and representation at consultant level, consistent with a ‘leaky pipeline’ in which disparity accrues after, rather than at, selection.^9^ While multifactorial in origin, these patterns reflect a sustained career progression and advancement, rather than in initial selection.^5^ These issues extend beyond workforce demographics, with direct implications for patient care. Similar trends are observed in trauma and burn care, where delayed presentation and lower referral rates contribute to poorer outcomes.^10^ Patients also report greater trust and satisfaction when treated by clinicians who understand their cultural background and lived experiences.^11^ Improving representation in PS is therefore not only a moral imperative, but essential to delivering equitable patient-centred care.

Although female representation in surgery has improved, gender imbalances persist.^6^ In 2023, women comprised 24% of PS consultants in the UK, up from 14% in 2010, this lags behind specialties such as ophthalmology and paediatric surgery.^6^^,^^12^ Despite increased entry into the specialty, progression to senior roles remains limited. Recognising the value of gender-concordant mentorship, RCSEng introduced the Women in Surgery Network and the Lady Estelle Wolfson Emerging Leaders Fellowship to dismantle stereotypes and promote leadership development.^13^ The persistence of the consultant gap reaffirms the need to align leadership pathways with the diversity of the training pipeline. Financial barriers also remain a barrier, where competitive portfolio requirements often demand substantial personal investment prior to formal training. Limited specialty exposure intensifies this burden, pushing candidates toward costly extracurricular activities including exams, courses, conferences, and higher degrees.^14^ In 2015, the estimated cost of demonstrating a clear commitment to PS was £15,334,^15^ adjusted for inflation and rising fees, this now exceeds £20,000.^14^ Although funding schemes exist, their reimbursement-based structure disproportionately disadvantages lower-income candidates, limiting access to formative opportunities and shaping long-term career trajectories.

This study explores the root causes of inequity in PS by examining how sociodemographic factors influence access to mentorship, research, and clinical exposure among UK medical students and pre-specialty residents. Acknowledging that disparities in progression are often reinforced by unequal access to guidance and formative opportunities, we aim to identify predictors of underrepresentation and differential attainment. By highlighting these barriers, we seek to inform the design of a targeted mentorship intervention that fosters clinical and academic engagement and promotes merit-based progression for those most disadvantaged.

## Methods & materials

### Participants

This national cross-sectional study adheres to the Strengthening the Reporting of Observational Studies in Epidemiology guidelines.^16^ Eligible participants included UK medical students (≥18 years) enrolled in the 2023/24 academic year,^17^ and postgraduate pre-specialty residents across 17 UK Foundation Schools.^18^ Exclusion criteria were prior discontinuation of medical education, current enrolment in PS specialty training, or inability to provide informed consent. Ethnic classifications followed the 2021 Census of England and Wales, as defined by the Office for National Statistics,^19^ and underrepresented groups were described according to UK government guidance on ethnicity nomenclature in research.^20^

### Procedure

A systematic review, conducted in line with PRISMA guidelines (Appendix, Supplemental Digital Content (SDC) 1, Tables 1 and 2), informed the design of a 20-item questionnaire hosted on Google Forms ™ (Appendix, SDC 2), exploring perceptions of EDI and mentorship in PS. Iterative feedback from regional leads, the study steering committee, and senior authors guided refinement. Recruitment followed a collaborative model via the UK Plastic Surgery Research Collaborative.^21^, ^22^ Between September and December 2024, regional leads from 41 of 46 UK medical schools and all 17 foundation schools disseminated the survey through institutional emails, social media, and student forums. Based on population estimates, a minimum of 380 responses per group was required to achieve representativeness with 95% confidence and a 5% margin of error. To ensure data integrity, the ‘Restrict to one response’ feature was enabled; duplicates were manually reviewed and excluded, and incomplete entries were automatically removed. As the survey was anonymous, General Medical Council or medical school registration numbers were not collected; eligibility and respondent type (medical student or pre-specialty doctor) were therefore self-declared. In addition to single-response restriction, manual review and exclusion of duplicates, and automatic removal of incomplete entries, self-reported institution, training stage, and demographic fields were cross-checked for internal consistency, and logically inconsistent or implausible records were excluded. Participants provided electronic informed consent after reviewing an information sheet outlining study aims, confidentiality, data use, and voluntary participation.

**Table 1:** Study characteristics.

Title	Sample Size	Intervention	Findings	Undergraduate Exposure	Curriculum and Teaching Preferences	Conceptions and Awareness	Influence of Mentorship and Clinical Exposure	Factors and Barriers to Pursuing Plastic Surgery
How to improve plastic surgery knowledge, skills and career interest in undergraduates in one day [C.R. Davis et al. Oct 2010]	121 medical students	One-day plastic surgery event	Significant improvement in knowledge, awareness, skills, and career interest post-course (p < 0.01). Medical students were more accurate at identifying plastic surgery procedures after the course (P < 0.001).	Not Reported	Not Reported	Medical students significantly improved in all four key themes (p < 0.01). 93 non-medical students completed questionnaires. Medical students were significantly more accurate at identifying plastic surgery procedures than non-medical students (P < 0.01), which was further strengthened after completing the course (P < 0.001).	Not Reported	Not Reported
Plastic Surgery Undergraduate Training: How a Single Local Event Can Inspire and Educate Medical Students [M. Khatib et al. Aug 2015]	39 medical students	Consultant-led lectures and workshops	Career interest increased by 12.5% (P < 0.0005). Improved perceptions of plastic surgery and significant enhancement of surgical skills, e.g., suturing (P < 0.0001).	Not Reported	Not Reported	Perception of the role of plastic surgeons in improving patient quality of life, increased by 18.31% (P = 0.063). B Before the course 10% of delegates perceived plastic surgery to be a superficial discipline and 20% perceived that plastic surgeons did not save lives. After completing the course, no delegates held those views.	Interest in pursuing a plastic surgery career significantly increased by 12.5% (P < 0.0005).	Not Reported
Burns teaching in UK medical schools: Is it enough? [R. Zinchenko et al. Feb 2016]	348 medical students	N/A - Cross-Sectional Study	1) 70% of students had no burns teaching, 90% lacked confidence in managing burns. 2) Universal agreement on the importance of burns management education.	1) 70% of the respondents have not received any specific teaching on how to manage patients with burns. 2) Nearly two-thirds of the students (66%) have never seen a patient being managed for burns	1) There was almost universal agreement about the importance of knowing how to manage a burn initially.	1) Poor Awareness: The majority of the respondents (57%) have not heard of the criteria for referring a burns patient for further specialist management.	Not Reported	Not Reported
				throughout their training.				
Factors influencing a career choice in plastic surgery as a UK medical student [S. Dean et al. June 2016]	153 medical students	N/A - Cross-Sectional Study	Career decisions influenced by early clinical exposure (53%- 69%) and extracurricular activities. Media negatively affected perceptions of plastic surgery. Year four students favoured lifestyle factors over clinical exposure.	82% of first-year students reported involvement in surgical skills such as suturing, with the least participation in fourth year (61%). 92% of students were aware of receiving formal teaching from a plastic surgeon early in the curriculum. 87% of students knew about plastic surgery LOCS, with an average of four to five students per year completing one or more. 76% were members of the student-led Swansea Surgical Society, with year two having the highest membership (84%).	40% rated anatomy teaching as good. The surgical society offered plastic surgery exposure through surgeon talks and hands-on surgical skill events (60% awareness). Students highlighted concerns about too much focus on breast and cosmetic surgery in the curriculum, particularly influenced by media portrayal (12% concern).	88% of students stated media portrayals would not influence their career choice, despite 74% to 91% agreeing that media projected plastic surgery negatively. Many students were unaware of the plastic surgery training pathway, with awareness lowest in year one (10% aware) and highest in year three (50% aware). Most students lacked knowledge of the competition ratio for plastic surgery training; only 19% recognized it as competitive.	All students completing plastic surgery LOCS found it influential in career decision-making, providing a "taster of the specialty." Mentorship was impactful in specific cases: a year-one student mentored by a hand surgeon pursued orthopaedics, and a year-three student with a plastic surgery mentor pursued the specialty. Clinical placements were reported as moderately to highly influential by first (28%), second (33%), and third (38%) years but less so by fourth years (43% stated slight influence).	First-year students showed the highest interest in a plastic surgery elective (45%), decreasing by the fourth year, where only three students expressed interest. Lifestyle considerations became more important for fourth-year students (33%), in contrast to earlier years valuing field experience. Career decisions were often made before medical school (68% of year-one students) or during early medical years (second year for 36% of fourth years).
How to train plastic surgeons of the future [C.R. Davis et al. Aug 2016]	175 medical students	Two national plastic surgery courses	Career interest increased after >75 h of exposure. Motivational factors included operative satisfaction (91%) and improving quality of life (89%).	Not Reported	Not Reported	Not Reported	A significant positive association was observed between the duration of plastic surgery exposure and career interest (Linear coefficient 0.12, 95% CI 0.08–0.17, P < 0.0001; r² = 0.15). 75 h was identified as a critical cut-off for categorizing students. Students with >75 h of exposure had a 31 percentage point higher mean career	Operative satisfaction (91%) Improving patient quality of life (89%) Interest in reconstructive surgery (88%)
							interest (89.2%, 95% CI 83.2–95.2) compared to those with ≤75 h (58.1%, 95% CI 52.9– 63.3) (P < 0.0001). 4) Influential Exposure Sources: Operating room experience, Consultant/Attending interactions, Resident interactions	
Improving Confidence and Practical Skills in Plastic Surgery for Medical Students and Junior Doctors: A One-Day Session. [C. Leung et al. Sept 2016]	35 participa nts: 6 core surgical trainees, 15 foundatio n doctors, and 14 medical students	One-day session covering knot tying, suturing, LA administratio n, and skin lesion excision	Significant improvement in confidence across all domains (p < 0.0001). Suturing (58.6%) and skin lesion excision (74.5%) showed the largest improvement. Course satisfaction rated at 9.13/10.	Not Reported	Not Reported	Overall course satisfaction was 9.13/10. Self-assessment confidence scores in all domains significantly improved after the course (p < 0.0001). Suturing (58.6%) and skin lesion excision (74.5%) demonstrated the largest improvement.	Not Reported	Not Reported
Plastic Surgery Inclusion in the Undergraduate Medical Curriculum: Perception, Challenges, and Career Choice [M. Farid et al. May 2017]	243 students	N/A - Cross-Sectional Study	Greater curriculum inclusion was preferred by McGill students (67%) vs. Birmingham students (49%). Awareness of plastic surgery significantly differed across institutions.	Not Reported	Teaching was the main source of knowledge for McGill students (39%, p < 0.001) while Birmingham students and control group chose the media (70%, p < 0.001). McGill students (67%) more than Birmingham (49%, p < 0.010) considered curriculum inclusion. Preferred learning method was lectures for McGill students (61%, p < 0.01) but an optional module for Birmingham (61%).	Poor Awareness: Questionnaire was analysed for 243 students (Birmingham, n = 171/332, 52%) (McGill n = 72/132, 54%). Birmingham students (14%) considered the word “plastic” synonymous with “cosmetic” more than McGill students (4%, p < 0.025).	A similar proportion (18%) from both student groups considered a career in plastic surgery.	Not Reported
Basic Burns Management E-Learning: A New	18 mediical students	E-learning tutorial on	72% of students expressed interest in e-learning modules.	While only a couple of students had received prior	1) 72% would be interested to have an e-learning module on basic	Not Reported	Not Reported	Not Reported
Teaching Tool [F.M. Egro July 2017]		burns management	2) High satisfaction with content clarity (78%−90%) and relevance to curriculum.	burns teaching, 50% of the cohort had an interest to pursue surgery as a career.	burns management in their medical curriculum. 2) Means of all domains specific to the e-learning were rated as “good” or “very good.” Students' rating for ease of use was 87%, usefulness was 88%, relevance to the medical curriculum was 90%, clarity and quality of content were 78% and 83%, respectively, design was 79%, and the overall satisfaction with this e- learning was 87%.			
The introduction of plastic and reconstructive surgery to the University of Glasgow undergraduate curriculum [G. Higgins et al. Feb 2020]	160 medical students	Teaching on plastic surgery subspecialties	81% believed plastic surgery was poorly represented. Post-teaching, knowledge significantly improved (p ≤ 0.00005). 3) Career interest increased slightly (39% to 41%).	129 (81%)) thought plastic surgery was poorly represented in their curriculum and wanted further information (98 (61%)).	129 (81%)) thought plastic surgery was poorly represented in their curriculum and wanted further information (98 (61%)).	1) Most and least recognised subspecialties were burns (48% (n = 75)) and perineal and lower limb reconstruction (0% (n = 0)), respectively, with more students identifying aesthetic surgery (16% (n = 26)) than hand (9% (n = 15)) or skin cancer surgery (6% (n = 9)).	1) Those interested in surgical careers increased from 39% (n = 63) to 41% (n = 66) with more males than females reporting interest (p ≤ 0.05).	Not Reported
Attitudes, Influences and Perceptions towards Plastic Surgery amongst Medical Students [T. Kidd et al. May 2021]	193 medical students	N/A - Cross-Sectional Study	56% of respondents identified private practice, reconstruction, and cosmetics with plastic surgery. Workshops and consultant-led teaching positively influenced perceptions. Lack of awareness of procedures was noted.	Not Reported	To overcome this lack of awareness and generate greater interest, students suggested greater plastics exposure, consultant-led teaching and workshops showcasing the specialty.	Phrases most strongly identified with plastic surgery included private practice, reconstruction and cosmetics. Placements, teaching staff and workshops/courses were found to influence perception of plastic surgery. Only 6% of students encountered antagonism towards plastic surgery encompassing themes of negative stereotypes of surgeons and connotations surrounding cosmetic surgery.	1) To overcome this lack of awareness and generate greater interest, students suggested greater plastics exposure, consultant-led teaching and workshops showcasing the specialty.	Not Reported
						4) Importantly, many students were largely unaware of the range of common procedures undertaken by plastic surgeons.		
Emergency burn education: Evaluating a surgical simulation-based intervention [S. Farzaneh et al. March 2023]	52 medical students	One-day course on burns management	Knowledge increased by 54.2% post-course (p < 0.01), with 31.4% rise in interest in plastic surgery. Sustained improvements observed 6 weeks post-course (knowledge +61%).	Not Reported	Not Reported	Students’ knowledge demonstrated a 54.2% increase in the post-course assessment (p < 0.01). 27% of participants (n = 14) participated in a 6-week post-course quiz. The knowledge assessment of these participants at 6 weeks post-course also demonstrated a sustained increase of 61.0% (p < 0.01).	Interest in plastic surgery was increased by 31.4% post-course (p < 0.01). At 6 weeks post-course, a sustained increase of 14.7% was demonstrated in comparison to pre-course data (p = 0.03).	Not Reported
A cross-sectional evaluation of the current state of wound healing education in the United Kingdom's undergraduate medical curriculum [Arwel T. Poacher et al. June 2023]	323 medical students	N/A - Cross-Sectional Study	Study identified limited clinical exposure, minimal teaching time, knowledge gaps in practical topics, lecture-dominant teaching format, preferred assessments and gaps and lack of confidence improvement.	68.4% of students reported receiving wound-related teaching during their undergraduate studies, but only 19.9% of this was integrated into clinical rotations within the core curriculum. Students reported an average of 2.25 h of preclinical and 1.00 hour of clinical-based teaching on wound management.	Wound education primarily focused on physiology and factors affecting wound healing, followed by: Wound infection identification (79.2%) Management of wound infections (78.2%) Wound assessment (66.1%) Chronic wound management (40.3%) Acute wound management (28.5%) Lecture-based teaching was the dominant format (83.3%), with smaller proportions receiving small group sessions (28.5%) or clinical placements (15.8%).	Knowledge gaps were identified in practical topics, particularly in: 1) Management of chronic and acute wounds 2) Types of wound dressings and differences between treating acute and chronic wounds (37.2% desired more education on this).	Limited dedicated clinical exposure (only 15.8% of students received wound education through clinical placements). No significant improvement in students' confidence to assess (R² = 0.190, p = 0.013), manage (R² = 0.060, p = 0.37), or prescribe for wounds (R² = 0.093, p = 0.18) over the course of undergraduate training.	Assessment preferences included multiple-choice questions on basic science (39.9%) and clinical scenarios (35.6%). 36.5% of students reported no formal assessment of wound healing knowledge during their undergraduate training.
Final year medical student confidence in assessing common hand injuries: Are we	260 medical students	N/A - Cross-Sectional Study	62.7% of final-year medical students (n = 163) reported no exposure to hand surgery.	1) 62.7% of final-year medical students (n = 163) reported no exposure to hand	1) 80.8% (n = 210) of students indicated interest in attending further courses or teaching sessions on hand injury assessment.	Not Reported	Not Reported	Not Reported
in safe hands? [U. Rehman et al. July 2023]			58.5% of students (n = 152) had no formal teaching in hand examinations as part of the core medical curriculum. Significant gaps exist in knowledge and skills related to hand injury assessment and surgical care due to lack of exposure and structured teaching. No specific mention of teaching formats, implying a possible lack of structured learning opportunities in hand surgery education. 80.8% (n = 210) expressed interest in attending courses or further teaching on the assessment of hand injuries. 15.4% (n = 40) responded "maybe," while only 3.8% (n = 10) responded "no."	surgery during their medical education. 2) 58.5% (n = 152) had no formal teaching on hand examinations as part of their core curriculum.	2) 15.4% (n = 40) responded "maybe," and only 3.8% (n = 10) were not interested in additional learning opportunities.			
Can We Support Second-Degree Students by Incorporating Non-Surgical Facial Aesthetics into Undergraduate Dentistry? [U. Rehman et al. July 2023]	114 dental students	N/A - Cross-Sectional Study	90% of respondents had no exposure to non-surgical facial aesthetics (NSFA). 76% were unaware of complications associated with dermal fillers. 3) Financial incentives motivated 52% to pursue NSFA, while lack of exposure deterred 47%.	A total of 103/114 (90%) of respondents reported no exposure to NSFA.	Not Reported	76% (87/114) and 75% (86/114) of students were not aware of the complications associated with the administration of dermal fillers and Botox injections, respectively. Regarding dermal filler reversal, we found most respondents, 93.0% (n = 106) were unable to identify the correct agent needed for reversal. Similarly, 84% (n = 96) of students were unaware of	Not Reported	24% (n = 27) of students strongly agreed they would consider pursuing a career in non-surgical facial aesthetics (NSFA). 44% (n = 50) agreed, 29% (n = 33) disagreed, and 4% (n = 4) strongly disagreed. 52% (n = 59) of students were attracted to NSFA
						the active ingredient found in dermal fillers.		due to financial incentives. 4) Most common deterring factor was lack of exposure, cited by 47% (n = 54) of students.
Are foundation doctors equipped with the skills necessary for the assessment and referral of a patient presenting with a suspected facial skin malignancy? [U. Rehman et al. Nov 2023]	142 foundatio n doctors	N/A - Cross-Sectional Study	50% of trainees had assessed ≤2 patients with facial skin cancer. 90% lacked confidence in performing a full skin examination. Confidence and knowledge gaps regarding facial skin malignancy management were evident.	Not Reported	Not Reported	Most FTs were not confident in performing a full skin examination (90.2%, 119/132), formulating differential diagnoses for a pigmented facial lesion (45.3%, 64/141) or investigating a facial SC (87.9%, 124/141).	Not Reported	Not Reported
CleftED: A National Collaborative Study of Undergraduate Education and Exposure to Cleft Lip and Palate [U. Rehman et al. Nov 2023]	3102 medical students	N/A - Cross-Sectional Study	44.3% of respondents had no exposure to cleft lip and palate (CLP) teaching. Despite this, 78.5% expressed interest in being involved in managing CLP patients in their future careers.	44.3% (n = 1374) of respondents had no exposure to CLP teaching up until their current year of education. 61.3% (n = 1903) of respondents had never been involved in the care of a CLP patient.	Not Reported	Not Reported	1) Despite lack of exposure, 78.5% (n = 2257) of respondents indicated a desire to be involved in the care of CLP patients in their future career.	Not Reported
Knowledge of Head and Neck Cancer Among Medical Students in the UK [R. Jaibaji et al. Feb 2024]	311 medical students	N/A - Cross-Sectional Study	23.2% of students reported receiving no teaching on head and neck cancer (HNC). 87.1% believed more HNC teaching was required.	Regarding HNC teaching, 72 students (23.2%) reported receiving no teaching at their medical school.	Of the 239 who reported receiving teaching, 169 (54.3%) received it in the format of a non-interactive, large group lecture. A total of 271 respondents (87.1%) believed that medical students at their university would benefit from more teaching on HNC.	Not Reported	Not Reported	Not Reported
Fostering innovation and sustainable thinking in surgery: an evaluation of a surgical hackathon [Z Ahmed et al. April 2024]	43 participa nts: 28 medical students, 6 foundatio n doctors, 3 core trainee, 4 registrars and 2 engineers	A surgical hackathon	65% of attendees were medical students, with doctors and engineers also participating. Delegates' confidence in approaching surgical innovation increased by 20% (p < 0.001). Key Issues Identified: Reducing waste packaging (70%), Promoting recyclable material usage (56%), Public perceptions of plastic surgery through social media (40%). Prize-Winning Initiatives: AI- enhanced operative pathway, Instrument sterilization improvements, Educational platform for teaching research and innovation skills.	1) 65% of hackathon attendees were medical students, demonstrating substantial student involvement.	Top three prizes were awarded for initiatives including: An AI-enhanced operative pathway. Instrument sterilization improvements. An educational platform for teaching research and innovation skills to students.	Participants identified key issues in surgery innovation Reducing waste packaging (70%). Promoting recyclable material usage (56%). Public perceptions of plastic surgery via social media (40%).	Not Reported	The hackathon significantly boosted participants' confidence in approaching surgical innovation (+20%, p < 0.001), which may encourage further engagement in plastic surgery and related fields.
PLAStic Surgery Teaching In the Undergraduate Curriculum of Medical Students -United Kingdom (PLASTICS-UK) [S. Gupta et al. June 2024]	2513 medical students	N/A - Cross-Sectional Study	56.3% of students had no formal teaching in plastic surgery. Breast reconstruction was the most recognized surgery (91.8%), while head and neck cancer surgery was the least recognized (35.9%). Non-curricular activities improved understanding and career aspirations.	1) Students perceived plastic surgery as challenging, competitive, and impacting quality of life; however, most had no formal teaching (56.3%); merely 6.2% had clinical exposure and 30.4% had an undergraduate plastic surgery placement available.	Not Reported	Breast reconstruction was the most frequently correctly identified surgery (91.8%), with head and neck cancer surgery the least (35.9%). Surgical aspirants were less likely to overestimate private practice (p < 0.001) but demonstrated greater self-confidence in plastic surgery understanding (p < 0.001) and their future ability to make plastic surgery referrals (p < 0.001), being more	Not Reported	Not Reported
						likely to pursue it (p < 0.001). 3) Surgical aspirants were 17.3% more likely to obtain non-curricular plastic surgery experience (p < 0.001), and 5.6% more self-confident dressing basic wounds (p < 0.001).		
Application of Problem-Based Learning Combined with Micro-Video Teaching in Burn Surgery [Z. Zhao et al. Sept 2024]	50 students	Problem-based learning combined with micro-video teaching	Observation group scored significantly higher on theory and skill tests than the control group (p < 0.05). Improved critical thinking ability and satisfaction with teaching methods.	Not Reported	Not Reported	Not Reported	Observation group possessed higher scores on theory tests and skill tests, greater critical thinking ability after teaching, higher evaluation of teaching methods and more satisfaction with their internships versus the control group (all p < 0.05).	Not Reported
Virtual Mentorship is a No-Brainer: The Application of a Virtual Mentorship Programme for Prospective Plastic Surgery Trainees [L.Z. Motie Nov 2024]	10 participa nts: 9 medical students, 1 foundatio n year doctor	Six virtual mentorship sessions	Significant increase in understanding of plastic surgery conditions, emergencies, and training pathways (p < 0.05). Interest in pursuing plastic surgery increased significantly. Programme received a 100% satisfaction rating.	Not Reported	Not Reported	Not Reported	There was a significant increase in the understanding of common plastic surgery conditions and emergencies (p < 0.05), as well as improved knowledge of the application processes for core surgical training (p < 0.05) and higher speciality training (p < 0.05). Interest in the speciality significantly increased (p < 0.05), and participants were more likely to seek in-person mentorship (p < 0.05). Programme was well-received, with 100% rating it as 'excellent' or 'very good’.	Not Reported
Broadening Horizons: A 3-Month Structured Webinar Series for Undergraduate and Postgraduate Plastic Surgery Education [L. Boyce et al. Dec 2024]	1008 participa nts	16-part webinar series exploring plastic surgery principles	96% satisfaction rate with the program, with significant improvements in knowledge (p = 0.01) and career interest maintained. Confidence in plastic surgery knowledge improved significantly from 2/5 to 3/5 (p = 0.01).	1) 63 (53–73) delegates, including undergraduate, postgraduate and international students, attended each lecture on average. 2) 75% of delegates felt they had previously received inadequate plastic surgery teaching.	Not Reported	Confidence in their knowledge of plastic surgery increased significantly from 2 (1–3) (not very confident) to 3 (2–5) (somewhat confident) (p = 0.01). Post-course exam scores increased significantly from 49% (32–67) to 56% (23– 85) (p = 0.05).	Career interest and positive perceptions were maintained.	Not Reported
The current state of undergraduate burn training within the UK: a national collaborative study. [U. Rehman et al. Dec, 2024]	950 medical students	N/A - Cross-Sectional Study	Out of the 950 responses received from 10 medical schools, 34% (n = 323) reported no burn-related instruction until the current academic year. 63.8% of students were not confident in assessing total body surface burns, 57.3% were not confident in initiating burn first aid management, and 69.5% were not confident in calculating fluid requirements for resuscitation. 84.2% felt that they had not received adequate teaching in burns during their undergraduate training.	1) 34% (n = 323) reported no burn-related instruction until the current academic year.	1) 84.2% felt that they had not received adequate teaching in burns during their undergraduate training.	1) 34% (n = 323) reported no burn-related instruction until the current academic year.	Not Reported	Not Reported

**Table 2:** ***Quality assessment & risk of bias*** Each study was individually reviewed for risk of bias using the Newcastle-Ottawa Scale (NOS), which was adapted to derive Agency for Healthcare Research and Quality (AHRQ) scores to assess study quality. (56).

Study	Selection	Comparability	Outcome	AHRQ Rating
Zinchenko et al. 2016	★ ★ ★	★	★	Poor
Farid et al. 2017	★ ★ ★	★	★ ★	Good
Higgins et al. 2020	★ ★ ★	★	★ ★	Good
Davis et al. 2016	★ ★ ★	★	★ ★	Good
Kidd et al. 2021	★ ★ ★	★	★ ★	Good
Dean et al. 2016	★ ★	★	★	Poor
Khatib et al. 2015	★ ★	★	★ ★	Fair
Davis et al. 2010	★ ★	★	★ ★	Fair
Leung et al. 2016	★ ★	★	★ ★	Fair
Francesco et al. 2017	★ ★ ★	★	★ ★	Good
Farzaneh et al. 2023	★ ★	★	★ ★	Fair
Arwel et al. 2023	★ ★ ★	★	★ ★	Good
Rehman et al. 2023 (clefted)	★ ★ ★	★	★ ★	Good
Rehman et al. 2023 (Facial Skin Malignancy)	★ ★ ★	★	★ ★	Good
Rehman et al. 2023 (Facial Aesthetics)	★ ★ ★	★	★ ★	Good
Motie et al. 2024	★	★	★★	Poor
Gupta et al. 2024	★ ★ ★	★	★ ★	Good
Zhao et al. 2024	★★	★	★★	Fair
Rehman et al. 2024 (Hand Injuries)	★ ★ ★	★	★ ★	Good
Boyce et al. 2024	★ ★ ★	★	★ ★	Good
Raian et al. 2024	★ ★ ★	★	★ ★	Good
Ahmed et al. 2024	★★	★	★★	Fair
Rehman et al. 2024 (Burns)	★★	★	★★	Fair

According to the conducted AHRQ rating, 14 studies were of good, 7 of fair and 3 of poor quality. (56) Of all 23 studies, 9 had evidence of selection bias, all had some evidence of comparability bias whilst 21 had some evidence of outcome bias and 2 had substantial evidence of outcome bias.

References

1. Khatib M, Soukup B, Boughton O, Amin K, Davis CR, Evans DM. Plastic Surgery Undergraduate Training: How a Single Local Event Can Inspire and Educate Medical Students. Annals of Plastic Surgery. 2015; 75 (2): 208. 10.1097/SAP.0000000000000058.

2. Zinchenko R, Perry FM, Dheansa BS. Burns teaching in UK medical schools: Is it enough? Burns. 2016; 42 (1): 178–183. 10.1016/j.burns.2015.10.003.

3. SAMERA DEAN MJ, NICK WILSON&nbsp, JONES. Factors influencing a career choice in plastic surgery as a UK medical student. General Plastic Surgery: 2016. https://www.thepmfajournal.com/features/features/post/factors-influencing-a-career-choice-in-plastic-surgery-as-a-uk-medical-student.

4. Davis CR, Trevatt AEJ, McGoldrick RB, Parrott FE, Mohanna P. How to train plastic surgeons of the future. Journal of Plastic, Reconstructive & Aesthetic Surgery. 2016; 69 (8): 1134–1140. 10.1016/j.bjps.2016.05.001.

5. Farid M, Vaughan R, Thomas S. Plastic Surgery Inclusion in the Undergraduate Medical Curriculum: Perception, Challenges, and Career Choice—A Comparative Study. Plastic Surgery International. 2017; 2017 9458741. 10.1155/2017/9458741.

6. Egro FM. Basic Burns Management E-Learning: A New Teaching Tool. Journal of Burn Care & Research. 2017; 38 (4): e715–e721. 10.1097/BCR.0000000000000462.

A precise sampling frame could not be defined due to stratified, decentralised dissemination; however, we estimated the target population using 2023–24 national data and recent studies on surgical career interest, yielding an estimated sampling frame of 14,093 (Appendix, SDC 3). The response rate was calculated as the proportion of valid responses within this group. A 20% threshold was adopted to minimise non-response bias; if not achieved, a structured non-responder analysis was conducted to assess representativeness. To further correct for over- or under-representation among key sociodemographic domains, post-stratification weighting adjustments were applied, ensuring that the weighted analysis better reflected the target population (Appendix, SDC 4)

### Measures

Outcome measures included: (i) interest in PS, assessed via a binary (yes/no) response; (ii) mentor type, categorised as a mentor in PS, a mentor from another specialty, or no mentor; (iii) perceived bias across racial, gender, and socioeconomic domains, measured using composite ordinal scales (1–5); and (iv) specialty considerations, including the influence of mentorship and EDI, similarly assessed on 1–5 scales. Composite variables for outcomes iii and iv demonstrated internal consistency (Cronbach’s alpha > 0.7). Predictor variables included: (a) demographic factors (gender, sexual orientation, ethnicity, respondent type, and disability status); (b) socioeconomic factors (household income, secondary school type, and familial links to medicine); and (c) academic factors (mentor availability, involvement in PS research, and clinical exposure). Age and year of study/training were excluded due to multicollinearity with respondent type, while geographical region (for doctors) and medical school (for students) were omitted to reduce redundancy.

### Statistical analysis

All analyses were conducted in Stata 17 (StataCorp, 2021). Categorical variables were summarised as frequencies and percentages; ordinal variables were reported as medians with interquartile ranges. Group differences were assessed using Wilcoxon rank-sum tests. Binary logistic regression was used for PS interest; multinomial logistic regression for mentor type; and ordinal logistic regression for perceptions of bias and specialty considerations. Cluster-robust standard errors accounted for clustering within medical and foundation schools to improve variance estimation. Predictor variables were dichotomised where appropriate to aid interpretability.

To assess disparities in specialty progression, survey responses on PS interest were compared with national PS resident demographics (ST3–ST8, 2023/24), obtained via a Freedom of Information request (Ref: FOI-2309-2021200). Proportional differences were assessed using chi-square tests, and logistic regression estimated the odds of progression to training. Crude and adjusted (adj.) odds ratios (ORs) were reported for binary and ordinal models, and relative risk ratios (RRRs) for multinomial models. Adjusted analyses included all predictor variables to minimise confounding. Sensitivity analyses compared results across the full sample and those expressing interest in PS. Multiple comparisons were controlled using the Benjamini-Hochberg procedure (p < 0.05), and missing data were handled via listwise deletion.

### Ethical approval

The study received ethical approval from the School Student Project Ethics Committee at from the Host University on the 20/9/2024.

## Results

### Demographics

Of 2608 responses, 61 incomplete and 20 duplicate entries were excluded, yielding 2528 valid responses (Table 3). Medical students comprised 81.1% (n = 2049) and doctors 18.9% (n = 479). Gender distribution mirrored UK undergraduate demographics: 53.7% (n = 1357) identified as women, 44.2% (n = 1117) as men, and 1.6% (n = 40) as other genders. 35.5% (n = 898) had attended comprehensive schools, 25.7% (n = 650) grammar schools, and 22.4% (n = 566) independent schools. Most respondents identified as heterosexual (85.5%, n = 2161), with 9.4% (n = 237) identifying as LGBTQ+. A total of 6.4% (n = 161) reported having a disability. Based on an estimated sampling frame of 14,093 individuals, the response rate was 17.9%. A non-responder analysis comparing respondents to UK training benchmarks from 2023 to 2024 showed broad representativeness across key sociodemographic domains (Appendix, SDC 5).

**Table 3:** Binary logistic regression (adjusted for clusters in universities and geographical regions) investigating the association between respondent characteristics and an in interest in plastic surgery.

Variable	Interested In Plastics?		Crude Estimates		Adjusted Estimates	
	*Yes (517)*	*No (2011)*	*OR (95% CI)*	*P-value^±^*	*OR (95% CI)*	*P-value^±^*
***Respondent Type***						
Student (2049)	389	1660	0.64 (0.45–0.91)	0.065	1.01 (0.67–1.50)	0.977
Doctor (479)	128	351	1 (Reference)		1 (Reference)	
***Gender Identity***						
Prefer not to say (14)	1	13	0.33 (0.37–2.82)	0.452	1.60 (0.23–10.87)	0.827
Female (1357)	291	1066	1.15 (0.88–1.49)	0.452	1.21 (0.92–1.60)	0.375
Other (40)	11	29	1.60 (0.75–3.40)	0.364	1.20 (0.51–2.81)	0.827
Male (1117)	214	903	1 (Reference)		1 (Reference)	
***Ethnicity***						
Prefer not to say (26)	1	25	0.14 (0.01–1.39)	0.266	0.22 (0.02–3.02)	0.448
Asian (1258)	224	1034	0.77 (0.55–1.09)	0.331	0.97 (0.67–1.40)	0.913
Black (233)	56	177	1.13 (0.71–1.80)	0.685	1.50 (0.98–2.28)	0.165
Mixed (208)	48	160	1.07 (0.77–1.49)	0.738	0.84 (0.59–1.22)	0.533
Other (159)	47	112	1.50 (0.87–2.58)	0.331	1.90 (1.04–3.47)	0.130
White (644)	141	503	1 (Reference)		1 (Reference)	
***Sexual Orientation***						
Prefer not to say (130)	22	108	0.82 (0.51–1.30)	0.553	0.96 (0.59–1.56)	0.913
LGBTQ+ (237)	64	173	1.48 (0.99–2.21)	0.182	1.45 (1.00–2.09)	0.152
Heterosexual (2161)	431	1730	1 (Reference)		1 (Reference)	
***Disability Status***						
Prefer not to say (79)	15	64	0.93 (0.49–1.75)	0.813	1.11 (0.68–1.80)	0.827
Yes (161)	40	121	1.31 (0.86–1.98)	0.364	1.06 (0.66–1.71)	0.894
No (2288)	462	1826	1 (Reference)		1 (Reference)	
***School Type***						
Prefer not to say (66)	14	52	1.24 (0.71–2.19)	0.576	1.46 (0.79–2.70)	0.427
Grammar (650)	127	523	1.12 (0.80–1.56)	0.611	1.24 (0.89–1.73)	0.408
Independent/Fee-paying (566)	141	425	1.53 (1.04–2.26)	0.128	1.69 (1.15–2.49)	0.039*
Outside UK (348)	75	273	1.27 (0.89–1.81)	0.358	1.40 (1.02–1.91)	0.130
Comprehensive (898)	160	738	1 (Reference)		1 (Reference)	
***Household Income***						
Prefer not to say (326)	43	283	0.48 (0.33–0.70)	<0.001^⁎⁎⁎^	0.52 (0.34–0.80)	0.021*
<£20,000 (224)	58	166	1.11 (0.63–1.96)	0.738	1.19 (0.61–2.33)	0.827
£20,000 - £39,999 (297)	62	235	0.84 (0.53–1.32)	0.576	0.91 (0.54–1.52)	0.837
£40,000 - £59,999 (384)	75	309	0.77 (0.52–1.14)	0.358	0.79 (0.52–1.20)	0.450
£60,000 - £79,999 (318)	45	273	0.52 (0.37–0.74)	<0.001^⁎⁎⁎^	0.56 (0.35–0.88)	0.061
≥£80,000 (979)	234	745	1 (Reference)		1 (Reference)	
***Medical Professional in Family***						
Prefer not to say (43)	5	38	0.47 (0.20–1.14)	0.266	0.48 (0.18–1.30)	0.343
Yes (1173)	226	947	0.86 (0.69–1.06)	0.331	0.70 (0.55–0.88)	0.019*
No (1312)	286	1026	1 (Reference)		1 (Reference)	
***Plastic Surgery Placement^+^***						
Yes (510)	186	324	2.93 (2.17–3.95)	<0.001^⁎⁎⁎^	2.11 (1.55–2.86)	<0.001^⁎⁎⁎^
No (2018)	331	1687	1 (Reference)		1 (Reference)	
***Plastic Surgery Research***						
Yes (288)	146	142	5.18 (3.40–7.89)	<0.001^⁎⁎⁎^	3.95 (2.70–5.78)	<0.001^⁎⁎⁎^
No (2240)	371	1869	1 (Reference)		1 (Reference)	
***Has a Mentor***						
Mentor - Other Specialty (562)	105	457	1.10 (0.80–1.51)	0.669	0.84 (0.59–1.21)	0.533
Mentor - Plastic Surgery (443)	148	295	2.39 (1.63–3.50)	<0.001^⁎⁎⁎^	1.31 (0.95–1.81)	0.265
No Mentor (1523)	264	1259	1 (Reference)		1 (Reference)	

OR = Odds Ratio, CI = Confidence Interval.

± Benjamini-Hochberg adjusted P-vales for 21 comparisons, + Including clinical placements, electives, taster weeks, and formal clinical rotations.

* Statistically Significant, ** Highly Statistically Significant, *** Very Highly Statistically Significant.

### Interest in plastic surgery

Of the 2528 respondents, 20.5% (n = 517) expressed interest in a career in PS (Table 3), with comparable interest among women (21.4%, n = 291) and men (26.4%, n = 214). Interest was generally higher among EM respondents than their White counterparts, though this was not statistically significant. Completing a PS rotation more than doubled the likelihood of interest (adj.OR 2.11, p < 0.001), while involvement in PS research nearly quadrupled it (adj.OR 3.95, p < 0.001). Having a PS mentor increased interest (OR 2.39, p < 0.001), although this effect was not significant after adjustment. Male applicants were significantly more likely than female applicants to progress to specialty training (OR 1.70, p < 0.001), while EM applicants were less likely to do so compared to White applicants (OR 0.43, p < 0.001).

### Mentorship in plastic surgery

After excluding 555 “Prefer not to say” responses, 1973 respondents were included in multinomial logistic regression (Table 4, SDC 3). Mentorship in PS was strongly associated with academic engagement, with mentees over nine times more likely to undertake PS research (adj.RRR 9.20, p < 0.001). Having a mentor in another specialty also increased research involvement (adj.RRR 3.14, p < 0.001) relative to having no mentor. Respondents from a medical family were more likely to have a mentor in another specialty (adj.RRR 1.44, p = 0.012). Among those interested in PS (n = 427), findings were consistent with the core analysis (Table 4, Table 5, SDC 3): specialty-concordant mentorship remained a strong predictor of PS research (adj.RRR 10.33, p = 0.002), while mentorship in another specialty also conferred increased engagement (adj.RRR 3.12, p = 0.010).

**Table 4:** Multinomial logistic regression – Entire Cohort.

Predictor	Mentor Type	Comparison Group	Reference Group	Crude RRR (95% CI)	P-Value	Adjusted RRR (95% CI)	P-Value
Respondent		Student (1,575)	Doctor (398)				

	Mentor - Plastic Surgery	252	106	0.51 (0.39–0.67)	<0.001	0.69 (0.50–0.94)	0.049
	Mentor - Other Specialty	358	86	0.89 (0.67–1.17)	0.509	1.04 (0.77–1.41)	0.800
No Mentor	965	206	Reference	–	Reference	–

Gender		Female (1,071)	Male (902)				

	Mentor - Plastic Surgery	179	179	0.82 (0.65–1.04)	0.162	1.19 (0.92–1.53)	0.353
	Mentor - Other Specialty	248	196	1.04 (0.83–1.29)	0.814	0.91 (0.73–1.14)	0.541
No Mentor	644	527	Reference	–	Reference	–

Ethnicity		Ethnic Minority (1,449)	White (524)				

	Mentor - Plastic Surgery	260	98	1.03 (0.79–1.35)	0.814	0.83 (0.62–1.12)	0.377
	Mentor - Other Specialty	346	98	1.37 (1.06–1.78)	0.036	0.70 (0.53–0.92)	0.033
No Mentor	843	328	Reference	–	Reference	–

Sexual Orientation		LGBTQ+ (201)	Heterosexual (1,772)				

	Mentor - Plastic Surgery	40	318	1.12 (0.77–1.64)	0.648	1.11 (0.72–1.70)	0.722
	Mentor - Other Specialty	43	401	0.96 (0.66–1.38)	0.814	1.12 (0.76–1.65)	0.681
No Mentor	118	1053	Reference	–	Reference	–

Disability		Yes (123)	No (1,850)				

	Mentor - Plastic Surgery	30	328	1.42 (0.91–2.21)	0.186	1.20 (0.94–1.54)	0.278
	Mentor - Other Specialty	22	422	0.81 (0.49–1.32)	0.509	0.91 (0.71–1.17)	0.579
No Mentor	71	1100	Reference	–	Reference	–

Household Income		<£80,000 (1,088)	≥£80,000 (885)				

	Mentor - Plastic Surgery	192	166	0.83 (0.66–1.06)	0.186	0.96 (0.73–1.28)	0.800
	Mentor - Other Specialty	215	229	0.68 (0.54–0.84)	<0.001	1.23 (0.97–1.57)	0.224
No Mentor	681	490	Reference	–	Reference	–

Secondary School		Independent School (692)	State School (1,281)				

	Mentor - Plastic Surgery	139	219	1.33 (1.04–1.70)	0.048	1.26 (0.96–1.66)	0.224
	Mentor - Other Specialty	174	270	1.35 (1.07–1.69)	0.025	1.15 (0.90–1.47)	0.389
No Mentor	379	792	Reference	–	Reference	–

Medical Family		Medical Family (923)	No Medical Family (1,050)				

	Mentor - Plastic Surgery	176	182	1.29 (1.02–1.64)	0.062	1.12 (0.85–1.48)	0.541
	Mentor - Other Specialty	246	198	1.66 (1.33–2.07)	<0.001	1.44 (1.13–1.83)	0.012
No Mentor	501	670	Reference	–	Reference	–

Plastic Surgery Research		Research (229)	No Research (1,744)				

	Mentor - Plastic Surgery	119	239	11.40 (7.95–16.35)	<0.001	9.20 (6.31–13.40)	<0.001
	Mentor - Other Specialty	61	383	3.65 (2.46–5.41)	<0.001	3.14 (2.09–4.72)	<0.001
No Mentor	49	1122	Reference	–	Reference	–

Plastic Surgery Placement		Rotation (416)	No Rotation (1,557)				

	Mentor - Plastic Surgery	135	223	3.49 (2.67–4.56)	<0.001	1.97 (1.44–2.70)	<0.001
	Mentor - Other Specialty	108	336	1.85 (1.42–2.43)	<0.001	1.60 (1.19–2.16)	0.010
No Mentor	173	998	Reference	–	Reference	–

Table 4: Multinomial logistic regression (adjusted for clusters in universities and geographical regions) investigating the association between dichotomised respondent characteristics and Mentor Type (Entire Cohort).

**Table 5:** Multinomial logistic regression – plastic surgery cohort.

Predictor	Mentor Type	Comparison Group	Reference Group	Crude RRR (95% CI)	P-Value	Adjusted RRR (95% CI)	P-Value
Respondent		Student (320)	Doctor (107)				

	Mentor - Plastic Surgery	81	41	0.45 (0.27–0.74)	0.008	0.95 (0.47–1.90)	0.883
	Mentor - Other Specialty	62	26	0.54 (0.30–0.96)	0.068	0.78 (0.39–1.59)	0.634
No Mentor	177	40	Reference		Reference	

Gender		Female (242)	Male (185)				

	Mentor - Plastic Surgery	63	59	0.83 (0.53–1.30)	0.520	1.27 (0.75–2.15)	0.588
	Mentor - Other Specialty	57	31	1.43 (0.86–2.39)	0.262	0.72 (0.42–1.24)	0.588
No Mentor	122	95	Reference		Reference	

Ethnicity		Ethnic Minority (307)	White (120)				

	Mentor - Plastic Surgery	77	45	0.55 (0.34–0.89)	0.040	1.21 (0.66–2.22)	0.634
	Mentor - Other Specialty	66	22	0.97 (0.55–1.72)	0.916	0.86 (0.45–1.65)	0.693
No Mentor	164	53	Reference		Reference	

Sexual orientation		LGBTQ+ (56)	Heterosexual (371)				

	Mentor - Plastic Surgery	14	108	0.78 (0.40–1.53)	0.547	0.69 (0.30–1.59)	0.588
	Mentor - Other Specialty	11	77	0.86 (0.41–1.79)	0.758	0.75 (0.33–1.75)	0.634
No Mentor	31	186	Reference		Reference	

Disability		Yes (29)	No (398)				

	Mentor - Plastic Surgery	8	114	1.10 (0.44–2.74)	0.880	1.16 (0.67–2.00)	0.664
	Mentor - Other Specialty	8	80	1.57 (0.63–3.93)	0.448	1.34 (0.80–2.24)	0.588
No Mentor	13	204	Reference		Reference	

Household income		<£80,000 (219)	≥£80,000 (208)				

	Mentor - Plastic Surgery	56	66	0.64 (0.41–0.99)	0.078	1.27 (0.71–2.27)	0.590
Mentor - Other Specialty	39	49	0.60 (0.36–0.98)	0.078	1.36 (0.76–2.44)	0.588
	No Mentor	124	93	Reference		Reference	

Secondary school		Independent (179)	State School (248)				

	Mentor - Plastic Surgery	52	70	1.27 (0.81–2.00)	0.424	1.32 (0.74–2.35)	0.588
	Mentor - Other Specialty	47	41	1.96 (1.19–3.24)	0.027	1.71 (0.96–3.04)	0.336
No Mentor	80	137	Reference		Reference	

Medical family		Medical Family (185)	No Medical Family (242)				

	Mentor - Plastic Surgery	62	60	1.81 (1.15–2.83)	0.029	1.41 (0.80–2.47)	0.588
	Mentor - Other Specialty	44	44	1.75 (1.06–2.88)	0.064	1.33 (0.76–2.34)	0.588
No Mentor	79	138	Reference		Reference	

Plastic surgery research		Research (118)	No Research (309)				

	Mentor - Plastic Surgery	72	50	13.44 (7.55–23.93)	<0.001	10.33 (5.59–19.09)	0.002
	Mentor - Other Specialty	25	63	3.70 (1.94–7.07)	<0.001	3.12 (1.57–6.21)	0.010
No Mentor	21	196	Reference		Reference	

Plastic surgery placement		Rotation (153)	No Rotation (274)				

	Mentor - Plastic Surgery	67	55	4.07 (2.53–6.55)	<0.001	2.12 (1.12–4.01)	0.133
	Mentor - Other Specialty	36	52	2.31 (1.36–3.93)	0.008	1.78 (0.93–3.43)	0.336
No Mentor	50	167	Reference	-	Reference	-

Table 5. Multinomial logistic regression (adjusted for clusters in universities and geographical regions) investigating the association between dichotomised respondent characteristics and Mentor Type (Subset of cohort Interested in Plastic Surgery).

### Perceptions of bias in plastic surgery

**Fig. 1:**
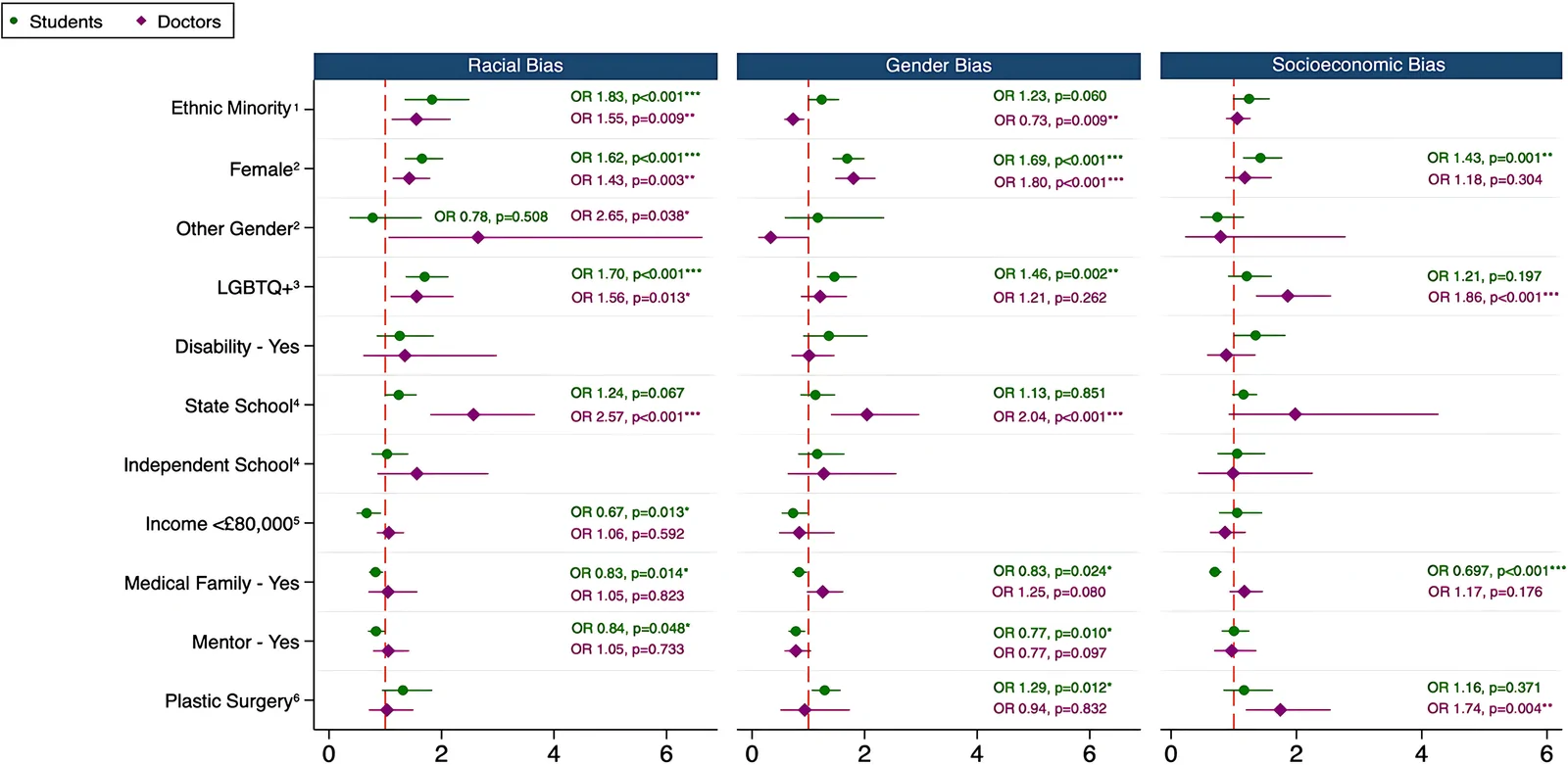
A forest plot of perceived racial, gender, and socio-economic bias in PS among medical students and doctors following ordinal logistic regression. Reference categories: 1) White, 2) Male, 3) Heterosexual, 4) School outside of the UK, 5) ≥£80,000, 6) Other Specialty.

Ordinal logistic regression showed that EM respondents were more likely to perceive racial bias in PS (students: adj.OR 1.83, p < 0.001; doctors: adj.OR 1.55, p = 0.009), as were women (students: adj.OR 1.65, p < 0.001; doctors: OR 1.43, p = 0.003) and LGBTQ+ respondents (students: adj.OR 1.70, p < 0.001; doctors: adj.OR 1.56, p = 0.013) (Fig. 1). Perceptions of gender bias were significantly higher amongst women (students: adj.OR 1.69, p < 0.001; doctors: adj.OR 1.80, p < 0.001) and students interested in PS (adj.OR 1.29, p = 0.012). Amongst students, mentorship was protective; reducing perceptions of racial (adj.OR 0.84, p = 0.048) and gender bias (adj.OR 0.78, p = 0.010). Socioeconomic bias was more frequently perceived by female students (adj.OR 1.43, p = 0.001) and LGBTQ+ doctors (adj.OR 1.86, p < 0.001), while doctors interested in PS were more likely to report socioeconomic bias than their peers (adj.OR 1.74, p = 0.004). In contrast, students from a medical family were less likely to perceive socioeconomic bias (adj.OR 0.69, p < 0.001). Additional group comparisons using Wilcoxon rank-sum tests are presented in SDC 7.

### Specialty considerations

**Fig. 2:**
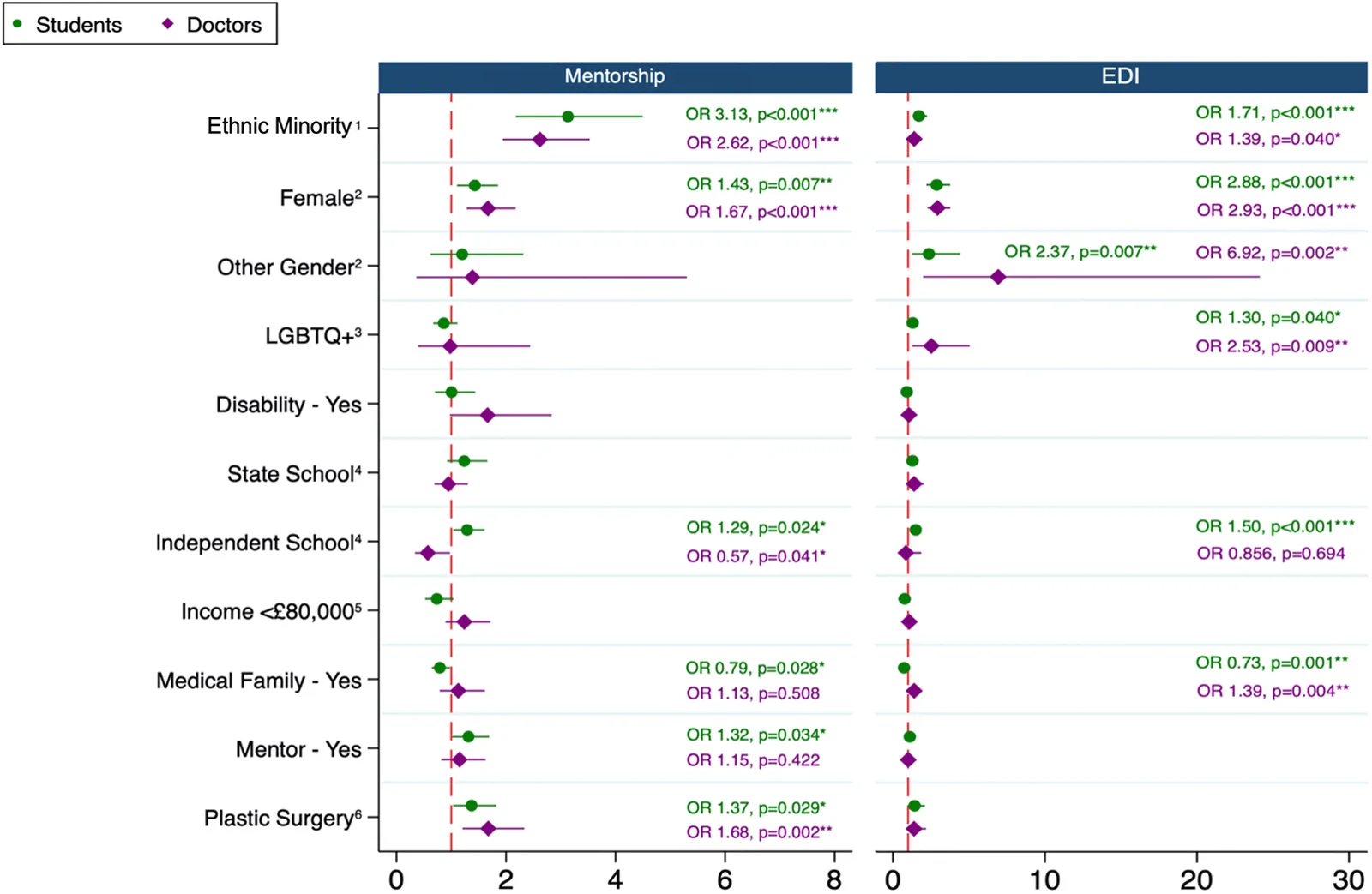
A forest plot of factors influencing the value placed on mentorship and EDI among medical students and doctors following ordinal logistic regression. Reference categories: 1) White, 2) Male, 3) Heterosexual, 4) School outside of the UK, 5) ≥£80,000, 6) Other Specialty.

EM respondents placed greater value on mentorship than White respondents (students: adj.OR 3.13, p < 0.001; doctors: adj.OR 2.62, p < 0.001), as did women (students: adj.OR 1.43, p = 0.007; doctors: adj.OR 1.67, p < 0.001) (Fig. 2). Mentored students were more likely to prioritise mentorship than those without a mentor (OR 1.32, p = 0.034), as were respondents interested in PS (students: adj.OR 1.37, p = 0.029; doctors: adj.OR 1.68, p = 0.002). Students from a medical family were less likely to value mentorship (adj.OR 0.79, p = 0.028), whereas those privately educated placed greater importance on it than their state-educated peers (adj.OR 1.29, p = 0.024). EM respondents also placed greater importance on EDI than White respondents (students: adj.OR 1.71, p < 0.001; doctors: adj.OR 1.39, p = 0.040). Additional comparisons using Wilcoxon rank-sum tests are detailed SDC 7.

## Discussion

To our knowledge, this is the most comprehensive evaluation of inclusivity and mentorship in PS among UK medical students and pre-specialty residents. Early clinical and academic exposure was a strong predictor of specialty interest. Despite comparable enthusiasm, EM individuals remain underrepresented in PS training, indicating systemic barriers beyond individual merit.^6^^,^^7^^,^^12^^,^^13^ Mentorship emerged as a key driver of progression, consistent with evidence linking it to higher publication output, specialty retention, and career satisfaction.^13^^,^^23^^,^^24^ It also played a protective role; reducing perceptions of exclusion, mitigating burnout, and fostering a sense of belonging.^24^

### Systemic barriers & challenges of modern mentorship

Differential attainment remains pervasive in surgery, disproportionately affecting EM candidates, who face lower appointability rates for surgical training,^8^^,^^25^ higher membership exam failure rates,^26^ and poorer outcomes in annual appraisals^25^ and consultancy applications.^6^, ^7^, ^8^ This group also report a perceived lack of institutional commitment to diversity in academia, which may deter research engagement.^23^^,^^27^ A ten-year review of leading PS journals found that EM authors comprised just 35% of senior authors, substantially fewer than their White counterparts.^23^ Notably, senior EM authors were significantly more likely to mentor junior colleagues from similar backgrounds, demonstrating the importance of identity-concordant mentorship in nurturing academic progression.^27^ Given the emphasis on research within ST3 recruitment, such mentorship is critical to dismantling barriers to academic access in PS.

Equally, addressing the intersection of socioeconomic background, professional access, and demographic factors is critical. Female PS residents face structural barriers around family planning, with nearly 60% delaying pregnancy due to concerns about training impact.^28^ Over half report negative attitudes from colleagues during pregnancy or maternity leave, and 41% receive no support on returning to work.^28^ Despite the procedural demands of PS, no structured return-to-surgery pathway or skill revalidation framework exists. Flexible working remains inconsistently applied, and the absence of institutional support for breastfeeding or childcare further compounds exclusion.^28^ These systemic shortcomings not only hinder progression but may actively dissuade women from entering or remaining in a specialty perceived as male dominated or incompatible with parenting. In contrast, students with a medical family member were less likely to perceive socioeconomic bias, likely benefiting from insider knowledge and tacit career guidance; privileges that mitigate the financial pressures of a surgical career. Similarly, privately educated students were more likely to prioritise mentorship, reflecting the structured guidance and early professional development embedded in independent education. These findings reveal the complex interplay of socioeconomic status and surgical training, reinforcing the need for targeted financial support and broader success metrics beyond demographic representation.

Mentorship must adapt to modern surgical training. Clinical, administrative, and academic pressures often constrain meaningful mentor–mentee relationships, while the underrepresentation of female and EM mentors limits access to relatable role models, reinforcing perceptions of exclusivity.^5^^,^^29^ Generational shifts have reshaped expectations, with younger residents prioritising work–life balance, mental health, and tailored guidance; values that diverge from traditional hierarchical models.^27^^,^^29^ Expanding access to mentorship for women, LGBTQ+ individuals, and first generation doctors is essential. These groups have historically faced socioeconomic exclusion, and improving access is key to dismantling structural barriers and ensuring that guidance is not limited to those with inherited social capital. Culturally responsive mentorship is essential to support equitable engagement and progression within an increasingly diverse surgical workforce.^30^

#### Grassroots mentorship: inspiring future plastic surgeons

Structured mentorship programmes enhance technical competence, shape professional identity, and encourage empathy and compassion among residents.^24^^,^^28^ Embedding mentorship into departmental routines requires robust institutional support to overcome the pressures of clinical and administrative workload.^29^ The RCSEng’s recent mentorship pilot for women and Specialty and Associate Specialist doctors signals a welcome shift towards addressing diversity gaps in surgical training.^13^ Specialty-specific provision has also emerged within the UK: the Plastic Surgery Trainees Association (PLASTA) runs a plastic surgery–specific mentorship scheme with over 150 enrolled mentees, and BAPRAS operates its own mentoring programme, complementing these college-level efforts. Importantly, mentorship adds value across the entire surgical career continuum, from medical student and pre-specialty trainee through to consultant and the years approaching retirement, and a life-course approach helps sustain engagement, leadership development, and the transfer of knowledge and experience at every stage. Targeted support for female surgeons in leadership and cultural competence training for mentors could help transform cultural differences from barriers into opportunities for growth and inclusion.^27^

Introducing mentorship early during undergraduate training empowers aspiring surgeons to navigate ethical challenges, build confidence, and pre-empt barriers before they are deeply embeded.^31^ In the UK, however, the paucity of targeted research and structured intervention continues to perpetuate inequities in PS. Other nations have advanced the discourse through studies on disparities in attainment, mentorship access, and career progression, particularly among IMGs and EM groups.^30^ These insights have informed national outreach, mentorship, and bias-reduction programmes that have improved representation across training and leadership.^29^^,^^32^^,^^33^ Although UK schemes such as the RCSEng, PLASTA, and BAPRAS mentoring programmes represent welcome progress, the relative scarcity of sustained, identity-concordant initiatives aimed specifically at pre-specialty trainees means these challenges are likely to persist without further investment, leaving the specialty short of a truly diverse and representative workforce. To address this, the authors piloted the INSPIRE mentorship programme between 2023 and 2024, a first-in-kind widening access initiative delivering longitudinal, identity-concordant mentorship to underrepresented students interested in PS. INSPIRE targets documented inequalities by offering meaningful research experiences and representative role modelling, strengthening students’ sense of purpose, promoting ethical integrity, and building lasting professional networks. The programme was endorsed by the RCSEng’s Grassroots in Surgery scheme, established in response to the Kennedy diversity review to promote inclusivity in surgery.^34^ Outcomes from the INSPIRE pilot have now been published, demonstrating improvements in students’ research output and interest in academic surgical careers.^35^ In 2026, the programme was expanded to students interested in all surgical specialties, with a focus not only on research but also on developing non-technical surgical skills.

### Strengths & limitations

This study benefits from its national scope, robust statistical methodology, and broad sampling across undergraduate and postgraduate training. However, several limitations warrant consideration. The cross-sectional design captures perceptions at a single time point, limiting causal inference and assessment of long-term mentorship effects. Longitudinal research is needed to evaluate whether mentorship influences specialty retention and leadership progression over time. Convenience sampling may have introduced selection bias, with overrepresentation of London-based institutions, as well as a study team and collaborative network themselves concentrated in London potentially limiting generalisability to all UK medical schools and training programmes. Survey distribution methods prevented calculation of response rates, raising the possibility of non-response bias if participants with strong mentorship experiences were more likely to respond. Self-reported data are also prone to recall and social desirability bias, particularly on EDI-related topics, where engagement may be overestimated or negative experiences underreported. While this study highlights disparities in mentorship access and career development, unmeasured confounders such as institutional culture, local mentorship initiatives, and personal motivation may also influence outcomes. Despite these limitations, the findings provide a critical foundation in understanding barriers to PS training and reinforce the need for structured, scalable mentorship interventions to promote equity in surgical careers.

## Conclusion

This study highlights the enduring impact of sociodemographic inequality on access to mentorship, research, and progression in PS. Structural barriers continue to constrain opportunity for underrepresented groups. Identity-concordant mentorship emerged as a powerful enabler of engagement, inclusion, and specialty readiness. While grassroots initiatives like INSPIRE offer a promising model, lasting change depends on institutional resolve: embedding mentorship into training, safeguarding protected time, and investing in inclusive, scalable frameworks. Achieving a surgical workforce that reflects the society it serves will require more than aspiration; it demands systemic change.

## Ethical approval

This study was reviewed and approved by the School Student Project Ethics Committee at Keele University on 20 September 2024. As a low-risk, anonymous survey of consenting adult medical students and doctors, the study was approved by this institutional research ethics committee; no separate Institutional Review Board (IRB) reference number was issued for a study of this category. All participants provided informed electronic consent, and the study was conducted in accordance with the principles of the Declaration of Helsinki.

## Declaration of competing interest

The authors do not have any conflicts of Interest and did not receive funding for this work

## Appendix Supplementary materials

Image, application 1

Image, application 2

Image, application 3

Image, application 4

Image, application 5
