# Supplementary material for: INSPIRE: Inclusivity in plastic surgery – Insights, recommendations, & education. A national cross-sectional study exploring perceptions of mentorship among aspiring plastic surgeons
Source: JPRAS Open. 2026 Jul 29;52:35–57. doi: 10.1016/j.jpra.2026.07.020 (PMC13553517; doi:10.1016/j.jpra.2026.07.020)
Supplement: Supplementary file 1 [file mmc1.docx]

Appendix, Supplemental Digital Content 1

Methods

A systematic review of the literature was conducted to evaluate common themes related to medical students' perceptions, awareness, and exposure to the specialty of plastic surgery. This review aimed to identify key factors influencing students' interest in the field, such as educational exposure, mentorship, and perceived barriers. The findings from the literature review were then used to inform the initial item generation for the study questionnaire, ensuring that the questions were grounded in existing evidence and reflective of the current trends and challenges within the specialty. This approach allowed for the development of a comprehensive and relevant questionnaire to assess students' experiences and attitudes towards plastic surgery. The PRISMA (Preferred Reporting Items for Systematic reviews and Meta-Analyses) guidelines were followed throughout this review. The search strategy and inclusion/exclusion criteria for the review is seen in Supplementary Tables S2 & S3.

| Databases | Search Strategy |
| --- | --- |
| MEDLINE®, Embase™, PubMed® and Google Scholar™ | (“medical student” OR “student” OR “undergraduate”) AND (“plastic surgery”) AND (“prepared” OR “confidence” OR “knowledge” OR “approach” OR “teaching method”) AND (“UK” OR “United Kingdom”) |

Search Strategy

| Inclusion Criteria | Exclusion Criteria |
| --- | --- |
| 1) Studies evaluating an undergraduate knowledge or skill-based plastic surgery   learning curriculum   2) Studies based in UK medical schools 3) Original and Primary Research 4) Studies published between 2003 and 2024 | 1) Research relating to participants other than   plastic surgery teaching   2) Studies not based in UK medical schools 3) Commentaries, abstracts, conference abstracts, letters to the editor 4) Studies published before 2003 |

Inclusion and Exclusion Criteria for Systematic Review.

Results

*Search results*

In total 478 papers were identified, and 60 full texts were reviewed. 23 papers fulfilled the inclusion criteria and were incorporated in this systematic review (Supplementary Figure S1) (1-24). There were five main themes: 1) undergraduate exposure, 2) curriculum and teaching preferences, 3) misconceptions and poor awareness, 4) influence of clinical exposure and mentorship on career interest and 5) motivational factors and barriers to pursuing PS.

PRISMA Flow Diagram.

*Study Characteristics*

13 papers focused on undergraduate exposure to plastic surgery whilst 11 focused on curriculum and teaching 17 regarding preconceptions and awareness, 12 on career interest and mentorship and 5 on barrier to PS. Across the studies gathered, 15 are cohort studies, 7 are national collaborative studies and 1 is a regional collaborative study (1-24).

Existing literature highlights several common barriers that deter individuals from pursuing a career in plastic surgery, such as the lengthy duration of training, concerns about work-life balance, and other related challenges. These factors are consistently cited across studies as significant obstacles influencing career choice. However, there is a notable gap in the research exploring how gender, socio-economic background, ethnicity, and misconceptions about the specialty impact interest and accessibility in plastic surgery. These underexplored areas are critical, as they may disproportionately affect certain groups and perpetuate inequities within the specialty. A study focusing on these barriers is necessary to better understand the nuanced challenges faced by underrepresented individuals and to identify actionable solutions that promote diversity, equity, and inclusion in plastic surgery. By addressing these factors, such research could help reduce disparities and ensure that the specialty is accessible and appealing to a broader and more diverse cohort of future surgeons.
