# Supplementary material for: INSPIRE: Inclusivity in plastic surgery – Insights, recommendations, & education. A national cross-sectional study exploring perceptions of mentorship among aspiring plastic surgeons
Source: JPRAS Open. 2026 Jul 29;52:35–57. doi: 10.1016/j.jpra.2026.07.020 (PMC13553517; doi:10.1016/j.jpra.2026.07.020)
Supplement: Supplementary file 2 [file mmc2.docx]

Appendix, Supplemental Digital Content 2

Inclusivity Study in Plastic Surgery: Insights, Recommendations & Education Introduction

This national cross-sectional study, led by a collaborative research group, explores the demographic makeup of plastic surgery aspirants, their perceptions of the specialty, and potential barriers to entry. Your participation in this short survey will help broaden our understanding of inclusivity within plastic surgery.

Survey Duration: ~5 minutes (20 questions)

Invitation to Participate

You are invited to take part in this study, which aims to explore inclusivity within plastic surgery training. Before deciding, please read the information below. If you have any questions, contact the research team.

Why is this research important?

Plastic surgery, like many surgical specialties, faces challenges in achieving diversity. Research has highlighted the underrepresentation of minority groups, which may discourage applications to the field. Key barriers include limited mentorship, financial challenges, and differential attainment. In the UK, there is a knowledge gap regarding the obstacles students and trainees face. Understanding these challenges can help inform policies and initiatives to improve access to the specialty.

Research Aims Primary Objective:

- Identify perceptual and structural barriers for medical students and junior doctors considering a career in plastic surgery.

Secondary Objectives:

- Analyse the demographics (gender, ethnicity, age, sexual orientation) of those interested in plastic surgery and compare them with national trainee data.
- Assess whether socioeconomic background (schooling, financial status) impacts perceptions of barriers to entry.
- Compare perceived barriers between medical students and junior doctors.
- Examine differences in barriers across demographic groups (ethnicity, gender, sexual orientation, religion).
- Investigate whether individuals from higher socioeconomic backgrounds have better access to mentorship, clinical exposure, and research opportunities.
- Identify differences in perceived barriers between those interested in plastic surgery and those considering other specialties.

Who Can Participate? Inclusion Criteria:

- Age 16 or above
- Enrolled in a UK medical degree
- Have not applied for, commenced, or completed a plastic surgery national training programme (NTN)

Exclusion Criteria:

- Under 16 years old
- Allied health professionals, non-medical students (e.g., physician associates, surgical nurse practitioners)
- Individuals who currently hold or have previously held a plastic surgery NTN

Do I have to take part?

Participation is voluntary. Choosing not to participate will not affect academic standing or career opportunities. The survey is anonymous, and once submitted, responses cannot be removed from the dataset.

What will happen if I take part?

You will complete an anonymous online survey (~5 minutes), covering perceptions, experiences, and insights on plastic surgery.

What are the benefits of taking part?

- Contribute to research: Responses will help identify and address barriers to entry.
- Inform future policies: Findings may guide educational and training policies.
- Reflect on experiences: The survey may help participants evaluate career aspirations and barriers.
- Access to results: A summary of findings will be shared.

What are the risks?

This is a low-risk, survey-based study.

How will my information be used?

- Publication: Findings will be published in academic journals.
- Access: A summary of results will be shared through university representatives.
- Confidentiality: Responses will be anonymised and presented in aggregate form.
- Future Research: Data may be used for further research under strict ethical standards.

Who will have access to my information?

- Data anonymity: No personal identifiers (e.g., names, emails, IP addresses) will be collected.
- Data security: Information will be stored in encrypted files on password-protected devices.
- Publication: Only anonymised, aggregate data will be shared.

What if I have concerns?

For any questions, please contact the research team.

For research governance concerns, contact the relevant institutional ethics board.

Consent

By submitting the survey, you confirm that:

- You have read and understood this information.
- You are 16 years or older and consent to participate.

Survey Questions

1. Which medical school are you currently enrolled in? * Mark only one option.
   - University of Aberdeen School of Medicine and Dentistry
   - Anglia Ruskin University School of Medicine
   - Aston University Medical School
   - Queen Mary University of London
   - University of Birmingham College of Medical and Dental Sciences
   - Brighton and Sussex Medical School
   - University of Bristol Medical School
   - University of Buckingham Medical School
   - University of Cambridge School of Clinical Medicine
   - Cardiff University School of Medicine
   - University of Dundee School of Medicine
   - Edge Hill University Medical School
   - The University of Edinburgh Medical School
   - University of Exeter Medical School
   - University of Glasgow School of Medicine
   - Hull York Medical School
   - Imperial College London Faculty of Medicine
   - Keele University School of Medicine
   - Kent and Medway Medical School
   - King's College London GKT School of Medical Education
   - Lancaster University Medical School
   - University of Leeds School of Medicine
   - University of Leicester Medical School
   - University of Liverpool School of Medicine
   - London School of Hygiene & Tropical Medicine
   - University of Manchester Medical School
   - Newcastle University School of Medical Education
   - University of East Anglia, Norwich Medical School
   - University of Nottingham School of Medicine
   - University of Nottingham- Lincoln Medical School
   - University of Oxford Medical Sciences Division
   - Plymouth University Peninsula Schools of Medicine and Dentistry
   - Queen's University Belfast School of Medicine
   - University of Sheffield Medical School
   - University of Southampton School of Medicine
   - University of St Andrews School of Medicine
   - St George's, University of London
   - University of Sunderland School of Medicine
   - Swansea University Medical School
   - University of Central Lancashire School of Medicine
   - University College London Medical School
   - University of Warwick Medical School
   - Brunel University London, Brunel Medical School
   - Ulster University, School of Medicine
   - University of Chester Medical School
   - Three Counties Medical School
2. What is your current year of study? * Mark only one option.
   - Year 1
   - Year 2
   - Year 3
   - Year 4
   - Year 5
   - Year 6
   - Intercalating Year
3. What is your age group? * Mark only one option.
   - Under 16
   - 16-24 years old
   - 25-34 years old
   - 35-44 years old
   - 45-54 years old
   - Over 55 years old
4. How do you describe your gender identity? * Mark only one option.
   - Male
   - Female
   - Non-Binary / Third Gender
   - Transgender Male
   - Transgender Female
   - Prefer not to say
5. How do you describe your ethnic background? * Mark only one option.
   - Black- African
   - Black- Caribbean
   - Other Black / African / Caribbean background
   - Asian / Asian British- Indian
   - Asian / Asian British- Pakistani
   - Asian / Asian British- Bangladeshi
   - Asian / Asian British- Chinese
   - Other Asian background
   - White and Black Caribbean
   - White and Black African
   - White and Asian
   - Other Mixed / Multiple ethnic background
   - White- English / Welsh / Scottish / Northern Irish / British
   - White- Irish
   - White- Gypsy or Irish Traveller
   - Other White background
   - Arab
   - Other ethnic background
   - Prefer not to say
6. How do you describe your sexual orientation? * Mark only one option.
   - Heterosexual or Straight
   - Gay or Lesbian
   - Bisexual
   - Prefer not to say
   - Other:
7. Do you consider yourself to have a disability? * Mark only one option.
   - Yes
   - No
   - Prefer not to say
8. What is your household’s total annual income before tax? * Mark only one option.
   - <£20,000
   - £20,001- £39,999
   - £40,000- £59,999
   - £60,000- £79,999
   - £80,000- £99,999
   - £100,000- £119,999
   - £120,000- £139,999
   - ≥£140,000
   - Prefer not to say
9. What type of school did you attend for most of your education between the ages of 11 and 16? * Mark only one option.
   - Comprehensive state-run or state-funded school
   - Grammar state-run or state-funded school
   - Independent or fee-paying school (Private School)
   - Prefer not to say
10. Do you have any family members who are medical professionals? * Mark only one option.
    - No
    - Yes
    - Prefer not to say
11. At present, which surgical specialty are you most interested in pursuing? * Mark only one option.
    - Plastic Surgery
    - Oral & Maxillofacial Surgery
    - ENT (Otolaryngology)
    - Neurosurgery
    - Paediatric Surgery
    - Cardiothoracic Surgery
    - General Surgery
    - Trauma & Orthopaedic Surgery
    - Urology
    - Vascular Surgery
12. How important are the following factors when considering a surgical specialty? * Mark only one option per row.

Factor	Not at all

important (1)

Low importance (2)

Neutral (3)

Important (4)

Very important (5)

Exposure to the specialty during medical school

○	○	○	○	○

Academic performance scores	○	○	○	○	○ Availability of mentorship from

individuals of the same racial/ethnic background

Availability of mentorship from individuals of the same gender identity

Racial/ethnic diversity within the specialty

○	○	○	○	○

○	○	○	○	○

○	○	○	○	○

Gender diversity within the specialty	○	○	○	○	○ Patient diversity within the specialty	○	○	○	○	○ Financial costs associated with training	○	○	○	○	○ Socio-economic background influence	○	○	○	○	○ Specialty competitiveness	○	○	○	○	○

13. To what extent do you agree or disagree with the following statements? * Mark only one option per row.

Strongly Disagree (1)

Statement

Somewhat Disagree (2)

Neither Agree nor Disagree (3)

Somewhat Agree (4)

Strongly Agree (5)

Plastic surgery has a higher proportion of male surgeons than female surgeons.

Plastic surgery has a higher proportion of White surgeons than surgeons from other ethnic backgrounds.

The financial cost of training in plastic surgery is high.

Training in plastic surgery is accessible to individuals from lower socio­economic backgrounds.

Plastic surgery provides mentorship opportunities from professionals of the same racial/ethnic background.

Plastic surgery provides mentorship opportunities from professionals of the same gender.

There is gender bias within plastic surgery training.

There is racial bias within plastic surgery training.

Plastic surgery serves a diverse patient population.

○	○	○	○	○

○	○	○	○	○

○	○	○	○	○

○	○	○	○	○

○	○	○	○	○

○	○	○	○	○

○	○	○	○	○

○	○	○	○	○

○	○	○	○	○

Strongly Disagree (1)

Statement

Somewhat Disagree (2)

Neither Agree nor Disagree (3)

Somewhat Agree (4)

Strongly Agree (5)

Educational content in plastic surgery is unintentionally biased towards a certain gender.

Educational content in plastic surgery is unintentionally biased towards a certain racial/ethnic group.

Safeguards are in place within the specialty to prevent bias and discrimination.

○	○	○	○	○

○	○	○	○	○

○	○	○	○	○

14. Have you participated in a plastic surgery clinical rotation during medical school? * Mark only one option.
    - Yes
    - No
15. Have you personally experienced any form of discrimination or bias during a plastic surgery placement, rotation, or taster week? *

Mark only one option.

- Yes
- No
- Not applicable

16. Have you personally witnessed any form of discrimination or bias during a plastic surgery placement, rotation, or taster week? *

Mark only one option.

- Yes
- No
- Not applicable

17. If you have not personally experienced or witnessed discrimination or bias in plastic surgery, do you think you might face this in the specialty? *

Mark only one option.

- Yes
- No
- Prefer not to say

18. Have you undertaken or are you currently involved in any research related to plastic surgery during medical school? *

Mark only one option.

- Yes
- No

19. Have you ever had a mentor in any specialty during medical school? * Mark only one option.
    - Yes – Plastic Surgery
    - Yes – Other Specialty
    - No
20. To what extent do you agree or disagree with the following statements? * Mark only one option per row.

Strongly Disagree (1)

Statement

Somewhat Disagree (2)

Neither Agree nor Disagree (3)

Somewhat Agree (4)

Strongly Agree (5)

Limited exposure to plastic surgery during undergraduate studies is a barrier to entry.

High academic achievement is important for entry into plastic surgery training.

The absence of mentors from the same racial/ethnic background is a barrier to career progression.

The absence of mentors of the same gender is a barrier to career progression.

A lack of gender diversity within the specialty is a barrier to career entry.

A lack of racial diversity within the specialty is a barrier to career entry.

The financial cost of training in plastic surgery is a barrier to entry.

Plastic surgery training appears to favour candidates from higher socio-economic backgrounds.

Perceived exclusivity within the specialty discourages applicants.

The rigorous nature of training in plastic surgery is a barrier to career entry.

Having a supportive professional network within the specialty is beneficial for career progression.

Lack of access to educational resources and additional plastic surgery experience is a barrier to entry.

○	○	○	○	○

○	○	○	○	○

○	○	○	○	○

○	○	○	○	○

○	○	○	○	○

○	○	○	○	○

○	○	○	○	○

○	○	○	○	○

○	○	○	○	○

○	○	○	○	○

○	○	○	○	○

○	○	○	○	○
