# Supplementary material for: INSPIRE: Inclusivity in plastic surgery – Insights, recommendations, & education. A national cross-sectional study exploring perceptions of mentorship among aspiring plastic surgeons
Source: JPRAS Open. 2026 Jul 29;52:35–57. doi: 10.1016/j.jpra.2026.07.020 (PMC13553517; doi:10.1016/j.jpra.2026.07.020)
Supplement: Supplementary file 3 [file mmc3.docx]

Appendix, Supplemental Digital Content 3

Response Rate Justification

*Study Context and Target Population*

This national cross-sectional study aimed to explore how sociodemographic factors influence access to mentorship, research, and clinical exposure in Plastic Surgery among UK medical students and pre-specialty doctors. The survey was designed for individuals actively considering a surgical career, in line with the study’s objective to inform the development of targeted widening access interventions within the surgical workforce.

Eligible participants included:

- Medical students (≥18 years) enrolled at Medical Schools Council-listed institutions (2023/24)
- Pre-specialty junior doctors: Foundation Years 1-3 and Core Surgical Trainees (CT1, CT2).

*Sampling Frame Estimation*

Given the absence of a defined sampling frame, we estimated the likely number of eligible respondents by:

1. Using published national government data to determine total cohort sizes in 2023–24
2. Applying recently published UK-wide estimates of the proportion of individuals expressing interest in surgical careers

| Group | Total (2023–24) | Interest in Surgery | Estimated Eligible |
| --- | --- | --- | --- |
| Medical Students | 42,000^1^ | 25%^2^ | 10,500 |
| Foundation Doctors (F1 + F2 + F3) | 15,700^3^ | 15%^4^ | 2,355 |
| Core Surgical Trainees | 1,238^5^ | 100% (by definition) | 1,238 |
| Total Eligible | 58,938 | - | 14,093 |

*Response Rate Calculation*

- Valid responses received: 2,528
- Estimated target population: 14,093

Response rate = (2,528 / 14,093) x 100 ≈17.9%

*References*

1. Lewis, J. (2023, September 12). *The cap on medical and dental student numbers in the UK*. House of Commons Library. https://commonslibrary.parliament.uk/research-briefings/cbp-9735/
2. O’Neill, L., Vassallo, A., & Dacre, J. (2023). Career intentions of medical students in the UK: A national, cross-sectional study. *BMJ Open, 13*(9), e075598. https://doi.dorg/10.1136/bmjopen-2023-075598
3. UK Foundation Programme Office. (2023). *2022 Recruitment Stats and Facts Report – FP Allocation extract*. Retrieved from https://foundationprogramme.nhs.uk/wp-content/uploads/sites/2/2024/04/2022%20Recruitment%20Stats%20and%20Facts%20Report_Allocati on%20by%20foundation%20school.pdf
4. Specialty Applications. (2024). *Core Surgical Training CT1 Competition Ratios*. https://www.specialty-applications.co.uk/competition-ratios/core-surgical-training-competition-ratios/
5. Health Education England. (2024). *2024 Competition Ratios - Medical Specialty Recruitment*. Retrieved from https://medical.hee.nhs.uk/medical-training-recruitment/medical-specialty-training/competition-ratios/2024-competition-ratios
