# Supplementary material for: INSPIRE: Inclusivity in plastic surgery – Insights, recommendations, & education. A national cross-sectional study exploring perceptions of mentorship among aspiring plastic surgeons
Source: JPRAS Open. 2026 Jul 29;52:35–57. doi: 10.1016/j.jpra.2026.07.020 (PMC13553517; doi:10.1016/j.jpra.2026.07.020)
Supplement: Supplementary file 4 [file mmc4.docx]

Appendix, Supplemental Digital Content 4

Weight Adjustment

Weight adjustments were applied to account for demographic over- and under-representation in the survey sample, ensuring that the analysis more accurately reflected the target population of UK medical students and pre-specialty residents. This was necessary because several demographic groups were either overrepresented (e.g., medical students) or underrepresented (e.g., pre-specialty residents) compared to national benchmarks. By applying weights, we corrected for these discrepancies, making the survey findings more generalisable and reducing the risk of bias in the results.

| Category | Expected Percentage | Observed Percentage | Weight |
| --- | --- | --- | --- |
| Medical Students | 73.3% | 81.1% | 0.904 |
| Pre-specialty Residents | 26.7% | 18.9% | 1.413 |
| Female | 60.0% | 53.7% | 1.117 |
| White | 47.0% | 55.5% | 0.847 |
| Comprehensive School | 56.0% | 35.5% | 1.577 |
| Low SES (<£40,000) | 5.0% | 23.7% | 0.211 |
| High SES (≥£60,000) | 75.0% | 58.9% | 1.273 |
