# Supplementary material for: INSPIRE: Inclusivity in plastic surgery – Insights, recommendations, & education. A national cross-sectional study exploring perceptions of mentorship among aspiring plastic surgeons
Source: JPRAS Open. 2026 Jul 29;52:35–57. doi: 10.1016/j.jpra.2026.07.020 (PMC13553517; doi:10.1016/j.jpra.2026.07.020)
Supplement: Supplementary file 5 [file mmc5.docx]

Appendix, Supplemental Digital Content 5

Non-responder Bias Analysis

| Demographic Category | UK Benchmark (2023/24) | Survey Respondents | Percentage Difference | Representation | Detailed Comments & Learnings |
| --- | --- | --- | --- | --- | --- |
| Training Stage |  |  |  |  |  |
| Medical Students | ~73.3%^1^ | 81.1% | +7.8 pp | Overrepresented | Higher student participation likely reflects early-career engagement. Future surveys might specifically boost outreach to  junior doctors to balance representation. |
| Pre-specialty Residents | ~26.7%^1,2^ | 18.9% | –7.8 pp | Underrepresented | Postgraduate doctors are underrepresented, a common issue in voluntary surveys. Consider tailored communication strategies to encourage  postgraduate involvement. |
| Gender |  |  |  |  |  |
| Female | ~60%^3,4^ | 53.7% | –6.3 pp | Underrepresented | Slight underrepresentation of women may reflect barriers or engagement issues. Ensure future communications  explicitly encourage female participation. |
| Male | ~40%^3,4^ | 44.2% | +4.2 pp | Aligned | Male respondents align well with national  benchmarks. |
| Other Gender | ~1%^4^ | 1.6% | +0.6 pp | Aligned | Slightly higher engagement possibly due to EDI theme. Continue inclusive messaging in future surveys to maintain  engagement. |
| Ethnicity |  |  |  |  |  |
| White | ~47%^5^ | 55.5% | +8.5 pp | Overrepresented | White respondents overrepresented; consider targeted outreach to ethnic minority trainee networks for future  surveys. |
| Asian | ~32%^5^ | 27.3% | –4.7 pp | Aligned | Good alignment; maintain current  strategies for engagement. |
| Black | ~5.5%^5^ | 5.8% | +0.3 pp | Aligned | Good alignment; maintain current  strategies for engagement. |
| Mixed | ~4.6%^5^ | 5.2% | +0.6 pp | Aligned | Slightly overrepresented; indicates  effectiveness of inclusive outreach. |
| Other | ~3.7%^5^ | 1.2% | –2.5 pp | Aligned | Slightly underrepresented; monitor  closely in future, but no immediate concern. |
| School Background |  |  |  |  |  |
| Comprehensive | ~56%^1^ | 35.5% | –20.5 pp | Underrepresented | Significant underrepresentation suggests potential barriers for state-schooled students. Increase outreach through  widening participation schemes. |
| Grammar | ~21%^1^ | 25.7% | +4.7 pp | Aligned | Good alignment; maintain current  strategies. |
| Independent | ~22%^1^ | 22.4% | +0.4 pp | Aligned | Good alignment; maintain current  strategies. |
| Disability |  |  |  |  |  |
| Disability | ~8%^6^ | 6.4% | –1.6 pp | Aligned | Slight underrepresentation, but no significant concerns. Ensure ongoing accessibility and confidential disclosure  mechanisms. |
| Sexual Orientation |  |  |  |  |  |
| LGBTQ+ | ~10%^6^ | 9.4% | –0.6 pp | Aligned | Close alignment indicates effective inclusive messaging. Continue explicit encouragement and anonymised  participation. |
| Household Income |  |  |  |  |  |
| Low (<£40,000) | ~5%^1^ | 23.7% | +18.7 pp | Overrepresented | Significant overrepresentation highlights survey’s resonance with lower-income trainees. Learnings suggest continued  focus on EDI is critical. |
| Middle (£40–£59,999) | ~20%^1^ | 17.4% | –2.6 pp | Aligned | Representative of middle-income backgrounds; maintain current  engagement strategies. |

| High (≥£60,000) | ~75%^1^ | 58.9% | –16.1 pp | Underrepresented | Underrepresentation of higher-income groups indicates potentially lower relevance of EDI themes for this cohort.  Consider varied messaging to increase  engagement. |
| --- | --- | --- | --- | --- | --- |

*Justification for 5pp Threshold:*

We employed a ±5 percentage point (5pp) threshold to identify meaningful differences between sample and population demographics in our non-responder analysis. This benchmark is supported by guidelines from the

U.S. Department of Education and the National Center for Education Statistics (NCES), which consider a 5pp difference as a criterion for significant demographic discrepancies.^7^

*References*

1. Medical Schools Council (2023). *Fostering Potential – 10 years on from Selecting for Excellence*. Retrieved from https://[www.medschools.ac.uk/news/new-report-shows-increase-in-students-from-disadvantaged-](http://www.medschools.ac.uk/news/new-report-shows-increase-in-students-from-disadvantaged-)backgrounds-attending-medical-school
2. UK Foundation Programme Office (2023). *2023 Foundation Programme Annual Report*. Retrieved from https://foundationprogramme.nhs.uk/publications/
3. Advance HE (2023). *Equality in higher education: students statistical report 2023*. Retrieved from https://[www.advance-he.ac.uk/knowledge-hub/equality-higher-education-statistical-reports-2023](http://www.advance-he.ac.uk/knowledge-hub/equality-higher-education-statistical-reports-2023)
4. University of Worcester (2023). *Equality, Diversity and Inclusion Annual Report 2023–24*. Retrieved from https://[www.worcester.ac.uk/documents/EDI-Annual-Report-2023-24.pdf](http://www.worcester.ac.uk/documents/EDI-Annual-Report-2023-24.pdf)
5. General Medical Council (2023). *The state of medical education and practice in the UK: Workforce report 2023*. Retrieved from https://[www.gmc-uk.org/about/what-we-do-and-why/data-and-research/the-state-of-](http://www.gmc-uk.org/about/what-we-do-and-why/data-and-research/the-state-of-)medical-education-and-practice-in-the-uk
6. University of Oxford (2023). *Equality, Diversity and Inclusion Report 2023–24*. Retrieved from https://edu.admin.ox.ac.uk/sites/default/files/edu/documents/media/equality_diversity_and_inclusion_rep ort_2023_24.pdf
7. Kastberg, D., Cummings, L., Lemanski, N., Ferraro, D., Perkins, R. C., Erberber, E., & Tsokodayi, Y. (2021). *U.S. Technical Report and User Guide for the 2018 Teaching and Learning International Survey (TALIS)* (NCES 2021-070). U.S. Department of Education. Retrieved from https://nces.ed.gov/pubs2021/2021070.pdf
